# Identification and validation of quantitative trait loci for ascites syndrome in broiler chickens using whole genome resequencing

**DOI:** 10.1186/s12863-020-00859-x

**Published:** 2020-05-20

**Authors:** Alia Parveen, Christa D. Jackson, Shatovisha Dey, Katy Tarrant, Nicholas Anthony, Douglas D. Rhoads

**Affiliations:** 1 Program in Cell and Molecular Biology, University of Arkansas, Fayetteville, AR 72701 USA; 2 Department of Biological Sciences, University of Arkansas, Fayetteville, AR 72701 USA; 3 Department of Biological Sciences, University of Arkansas, Fayetteville, AR 72701 USA

**Keywords:** broiler, ascites, quantitative trait locus, CPQ, LRRTM4, hypertension, genome

## Abstract

**Background:**

Ascites syndrome is a hypertensive, multifactorial, multigene trait affecting meat-type chickens imposing significant economic losses on the broiler industry. A region containing the CPQ gene has been previously identified as significantly affecting ascites phenotype. The region was discovered through whole genome resequencing focused on chicken chromosome 2. The association was confirmed through further genotyping in multiple broiler populations.

**Results:**

The whole genome resequencing analyses have now been extended to the current chicken genome assembly. DNA samples were pooled according to gender and phenotype and the pools subjected to next generation sequencing. Loci were identified as clusters of single nucleotide polymorphisms where frequencies of the polymorphisms differed between resistant and susceptible chickens. The chickens are an unselected line descended from a commercial elite broiler line. Regions identified were specific to one or both genders. The data identify a total of 28 regions as potential quantitative trait loci for ascites. The genes from these regions have been associated with hypertensive-related traits in human association studies. One region on chicken chromosome 28 contains the LRRTM4 gene. Additional genotyping for the LRRTM4 region demonstrates an epistatic interaction with the CPQ region for ascites phenotype.

**Conclusions:**

The 28 regions identified were not previously identified in a multi-generational genome wide association study using 60k Single Nucleotide Polymorphism panels. This work demonstrates the utility of whole genome resequencing as a cost effective, direct, and efficient method for identifying specific gene regions affecting complex traits. The approach is applicable to any organism with a genome assembly and requires no *a priori* assumptions.

## Background

Pulmonary hypertension syndrome (PHS) or ascites syndrome (AS) manifests as a complication of selection for rapid growth and high meat yield in broilers. AS is associated with accumulation of fluid in the abdominal cavity leading to death [1–6]. AS is a pathophysiological progression in which rapid growing birds are unable to meet the high oxygen (O_2_) demand for elevated metabolic rates [1–3, 6–8]. The hypoxic condition stimulates cardiac output leading to constriction of pulmonary arterioles causing hypertension in pulmonary circulation, right ventricular hypertrophy, and eventually right ventricular failure [3, 6, 9]. The primary cause of this disease is unknown, which makes it idiopathic in nature and makes AS an excellent model for human idiopathic arteriole hypertension [6, 10, 11]. AS incidence is influenced through environmental conditions including ventilation [12], lighting [13], temperature [14], feed restriction [1, 15], and diet composition [16–18]. However, world-wide economic losses are still estimated to be around 100 million dollars per year [6] (personal communication from Cobb-Vantress Inc. 2015). Many have suggested a genetic component to PHS, since AS related traits such as cardiac hypertrophy, and abdominal fluid, have moderate to high heritabilities [19–27]. Identification of quantitative trait loci (QTL) specifically affecting AS could lower economic losses in the broiler industry, with minimal impact on growth rate or meat yield.

Multiple Single Nucleotide Polymorphism (SNP) panel based genome wide association studies (GWAS) were used to identify potential genetic markers on chromosome 1, 2, 4, 9, and Z for this disease [28–33]. Unfortunately, further genotyping or marker assisted selection have only shown a marginal/minimal association between these regions and AS (unpublished). A whole-genome-resequencing (WGR) approach to further investigate potential regions on chromosomes 2 and 9 [29] did not support the previous regions but did identify a new region on chromosome 2 (127.65 – 127.75 Mbp; November 2017 assembly 5 coordinates) spanning part of the gene for plasma glutamate carboxypeptidase (PGCP or CPQ). GWAS data associates this gene with electrocardiogram, hypertension and blood pressure in humans (https://www.ncbi.nlm.nih.gov/gap/phegeni). Additional genotyping in a broiler research line, and three commercial broiler lines, demonstrated an association of homozygotes for the non-reference SNPs in this region with resistance in male birds. The CPQ gene region is the first demonstrated marker for resistance to AS in broilers [34]. The WGR investigation has now been extended to the rest of the genome to identify 27 additional regions as potential Quantitative Trait Loci (QTLs) for AS. Additional genotyping for one these new regions (LRRTM4) confirmed an association with AS. In addition, the data support a strong epistatic interaction between LRRTM4 and CPQ in contributing to AS phenotype.

## Results

### Whole Genome Resequencing and Templated Assembly

**Fig. 1:**
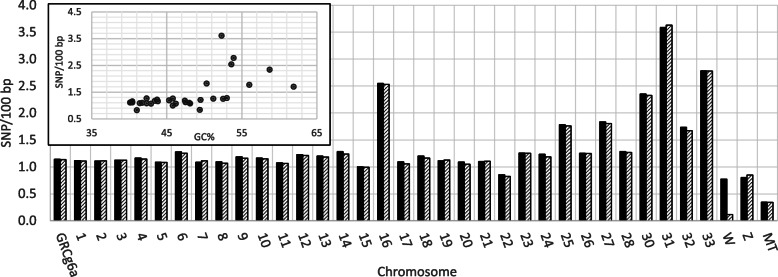
SNP density plots by chromosome in REL line birds. The SNP density computed per 100 bp is plotted for the entire genome (GRCg6a) or each individual chromosome for male (solid) and female (hatched). GRCg6a is for the entire genome and MT is mitochondrial genome. The insert is a plot of SNP density by GC% for individual chromosomes (excludes mitochondrial genome)

Next-generation WGR data was generated for eight pools of 10 individual DNA samples representing two biological replicates for each gender for both AS resistant and susceptible birds from the unselected, Relaxed (REL) line. The REL is descended from an elite commercial line [35]. Each pool was sequenced to >66x coverage using paired-end (2 x 125-bp) Illumina sequencing. FASTQ reads for each pool were mapped onto the May 2018 GRCg6a reference genome. The average read counts for all pools was 534,902,802±6,325,923, with 93.4±0.2% reads successfully assembled onto the reference genome. Potential SNPs identified by the read mapping totaled 12,024,469 in male pools and 11,933,041 in female pools. SNP counts were recorded, and SNP densities plotted for each chromosome according to gender (Fig. 1). The SNP density was observed to be higher for some of the microchromosomes in the assemblies for both genders. Whereas the average chromosomal SNP density is 1.36±0.61, SNP densities for microchromosomes 30, 31 and 33, are 2.3 to 3.6 SNPs per 100 bp. Most chromosomes have GC content of ≤ 47%. Chromosomes 30, 31 and 33 have a GC% of 58.7, 52.3, and 53.9, respectively. The current assembly of chromosome 30 is 1.82 Mbp, while chromosomes 31 and 33 are more complete at 6.15 and 7.82 Mbp, respectively. However, the assembly for chromosome 28 is 5.12 Mbp at 53% GC and the SNP density is 1.276/100 bp. This pattern of SNP density by chromosome is consistent with that observed for 3 commercial lines (manuscript in preparation) so it is not unique to the REL line. The SNP density is also not a function of read depth as read depth was roughly equivalent across all the chromosomes.

**Fig. 2:**
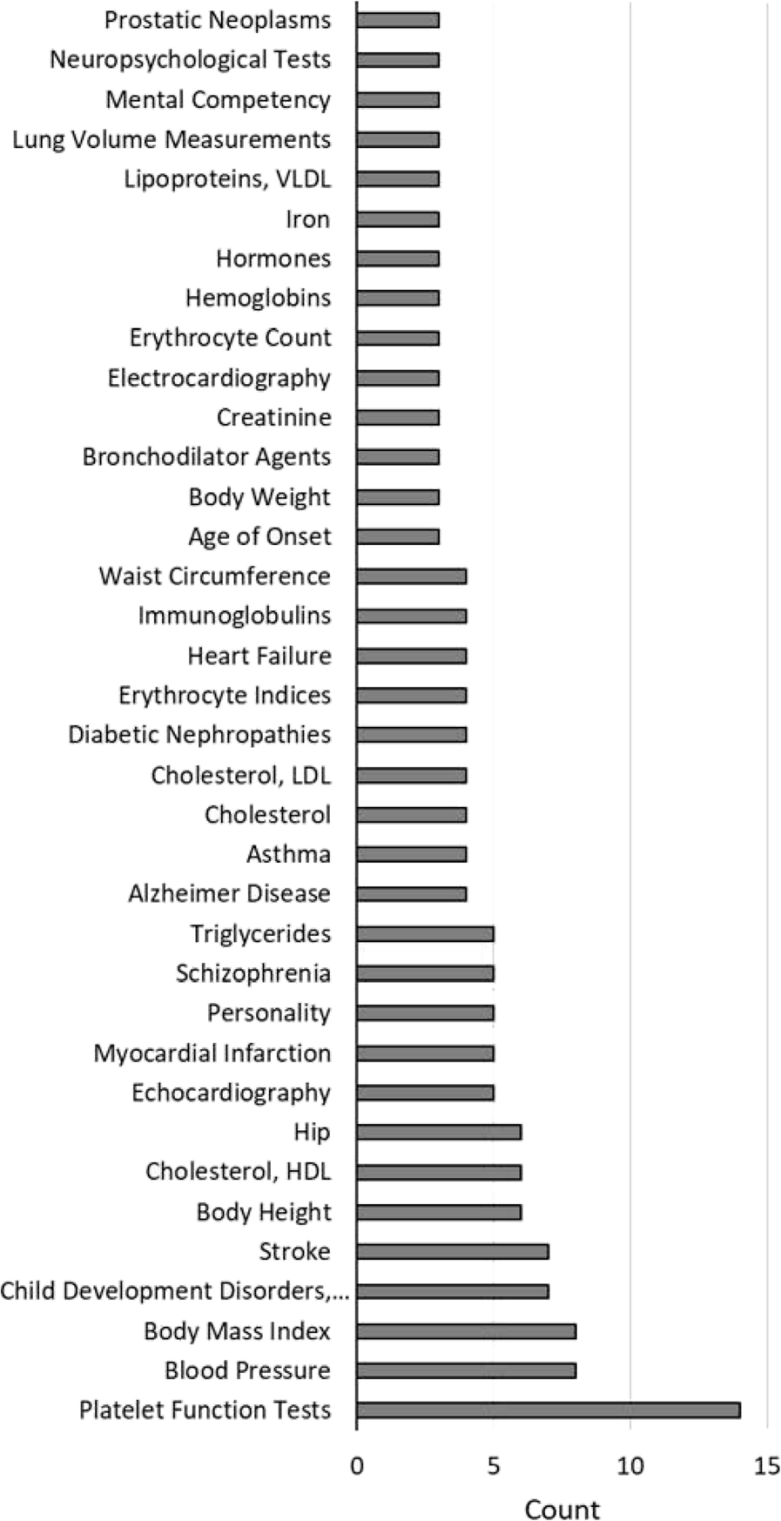
Human phenotypic traits associated with genes found in the potential QTL regions. Chicken gene names from the regions listed in Table 1 were used to search the human PheGenI database at NCBI**.** Phenotypes identified were then used to total the number of regions which were associated with that trait in human GWAS studies. Only traits associated with 3 or more regions are listed

To identify potential QTLs for AS phenotype, the difference between the frequency of the SNP in resistant versus susceptible phenotypes were calculated (resistant SNP frequency - susceptible SNP frequency). The SNP frequency differences were visually inspected using the Integrative Genomics Viewer (IGViewer [36];) according to chromosome and chromosomal location for both genders to visually identify clusters of SNPs with frequencies skewed with respect to phenotype for either or both genders. Specifically, the SNP frequencies were scanned for clusters of SNPs showing frequency differences of >20%. Twenty eight regions on 13 chromosomes were detected showing clusters of SNPs differentially represented in the different phenotypes (Table 1). The identified regions included 15 that showed association in both genders, while 8 appeared to be specific to males and 5 to females. The majority of regions showed a higher frequency of non-reference SNPs in the ascites resistant birds, with 8 associated with resistance in both genders, 5 male specific and 3 female specific. The non-reference SNPs were associated with susceptibility (higher frequency in susceptible) for 1 region in both genders, 3 male specific and 2 female specific. There were two regions where the non-reference SNPs were associated with resistance in males and with susceptibility in females, while there were four regions where the reverse was found. The 64 genes identified in these regions (Table 1), were used to search the NCBI Phenotype-Genotype Integrator, (PheGenI; https://www.ncbi.nlm.nih.gov/gap/phegeni) for phenotypes that had been identified in human GWAS as associated with traits possibly contributing to PHS or AS. The most frequent traits from the PheGenI output are presented in Fig. 2. Traits that are of particular interest regarding AS in broilers include: platelet function tests, blood pressure, body mass index, echocardiography, mycocardial infarction, erythrocyte indices, and heart failure. Thus, the human GWAS data supports these 28 regions as potential candidate QTLs for AS.

**Table 1:** Potential QTL regions for ascites syndrome based on WGR in the REL line

	Mbp			Res-Sus SNP frequency		
Chr	Start	Stop	Size	Male	Female	Genes within Region
1	48.18	48.41	0.23	30%	30%	APLD,GPRC5A,HEBP1,FAM234B,GSG1, EMP1,MIR6581
1	170.483	170.53	0.05	40%	0%	CAB39L
1	175.68	175.87	0.19	-25%	30%	PDS5B
1	182.31	182.46	0.15	40%	20%	AASDHPPT,KBTBD3,MSANTD4
1	183.65	183.95	0.30	35%	35%	DCUN1D5,MMP13,MMP10,MMP3,MMP7,BIRC2
2	22.86	23.03	0.17	50%	0%	SAMD9L,HEPACAM2,VP50
2	34.47	34.61	0.14	40%	-20%	PLCL2
2	91.85	91.92	0.07	50%	0%	CNDP2,FAM69C
2	95.14	95.22	0.08	-30%	0%	CDH19
2	122.75	122.83	0.08	45%	-25%	CA2
2	126.97	127.09	0.12	40%	20%	CPQ**
3	37.27	37.36	0.09	-30%	40%	RYR2
3	48.98	49.00	0.02	-20%	30%	RMND1
3	50.41	50.44	0.03	40%	0%	SCAF8
3	100.70	101.10	0.40	0%	-30%	OSR1
4	36.72	36.90	0.18	40%	0%	GRID2
5	13.26	13.29	0.03	0%	50%	DDTNFR23, CARS
5	25.44	25.47	0.04	-50%	25%	SPTBN5
6	28.82	28.87	0.05	0%	40%	ABLIM1
10	6.49	6.54	0.05	40%	30%	TJP1
14	1.48	1.66	0.18	0%	30%	LMTK2,BHLHA15,TECPR1,BRI3,BAIAP2L1, NPTX2
20	9.06	9.10	0.04	0%	-40%	PXDNL,PCMTD2
22	4.40	4.48	0.08	60%	20%	LRRTM4
27	7.85	7.98	0.13	-30%	0%	RAMP2,WNK4,COA3,BECN1,PSME3,AOC3, G6PC,PTGES3L,RPL27,IFI35,VAT1,RND2
28	0.59	0.63	0.05	-25%	0%	TIMM44,HNRNPM
Z	18.60	18.73	0.13	-25%	-50%	PDE4D
Z	19.10	19.50	0.40	25%	50%	ZSWIM6,KIF2A
Z	33.87	33.90	0.03	25%	25%	SLC24A2

Regions are listed by Chromosome (Chr) and Megabase pair (Mbp) Start, Stop and Size, in the chicken genome GRCg6a assembly. Res-Sus SNP frequency is the difference in the approximate maximum frequency difference for the non-reference SNP between the phenotypes of the indicated genders. Positive Res-Sus SNP frequency difference indicates the non-reference SNPs are associated with resistance, negative values indicate association with susceptibility. **Gga2 region previously verified as a QTL [34]

### LRRTM4 Genotyping in Association with Ascites Syndrome

**Fig. 3:**
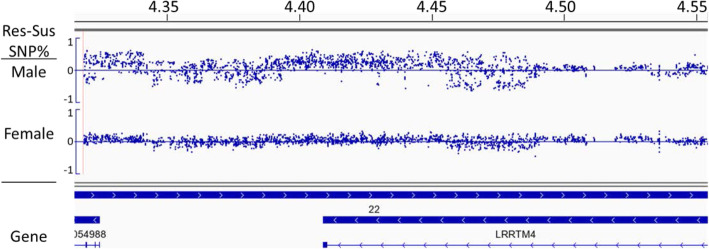
Integrative Genomics Viewer display of SNP frequency difference plots for a possible QTL for ascites syndrome on chromosome 22. Difference in SNP frequency for the alternative phenotypes are plotted according to position (Mbp). Resistant minus Susceptible SNP frequency (Res-Sus SNP frequency) are for the male and female data aligned with the gene annotations showing an association with the 3’ end of the LRRTM4 gene

The region on chromosome 2 for CPQ has already been extensively analyzed for association with ascites phenotype and confirmed for association of the non-reference SNPs with ascites resistance in males [34]. The region on chromosome 22 associated with the LRRTM4 gene was selected for further investigation because an association was found in both genders, with a larger frequency difference in males than females. This region contains 711 successive, high-quality SNPs spanning the 4.400-4.455 Mbp region on Gga22 (Fig. 3). The frequency difference (resistant SNP frequency - susceptible SNP frequency) averages 27% in males and 7% in females. In males 641 SNPs have a positive SNP frequency difference while only 11 are negative. In females there were 261 positive, and 12 negative, for SNP frequency difference. Of these SNPs, 560 covered 45.1 kbp of the 3’end of the LRRTM4 gene which spans 211.6 kbp from 4,409,156 to 4,620,835 Mbp. The LRRTM4 gene encodes the leucine-rich repeat transmembrane neuronal protein 4 which has been suggested to play a role in the regulation of dendritic spine development in the nervous system [37, 38]. NCBI PheGenI associates the human LRRTM4 gene region with traits such as antihypertension, carotid artery disease, coronary heart disease, and pulmonary embolism, supporting a probable association with AS in broilers.

An exonuclease assay was developed to target two SNPs (4,405,679, and 4,405,681 Mbp) which gave SNP frequency differences of approximately 50% in males and 15% in females. These SNPs are in the intergenic region, 3.5 kbp from the 3’ end of the LRRTM4 gene. The exonuclease assay was used to genotype more than 600 archived DNA samples from REL birds previously phenotyped for AS susceptibility in the hypobaric chamber [29, 33, 34]. The observed genotype frequencies were in agreement with calculated genotype frequencies (computed from allele frequencies) consistent with Hardy Weinberg Equilibrium (HWE) which implies that the quantitative Polymerase Chain Reaction (qPCR) genotyping is valid, there are no significant issues with null alleles, and that the samples of DNA utilized was non-biased. These same DNA samples had been used in the genotype associations with AS for the CPQ gene on chromosome 2 [34]. Based on the WGR SNP frequency plots the expectation was an association of resistance for males homozygous with the non-reference SNPs. Instead there was a significant association of the homozygous non-reference SNPs in females (AG genotype in females in Table 2; adjusted *P*-value=0.047). There was also a significant adjusted *P*-value (*P*=0.0083) for all samples, combining male and female data (Table 2). In both cases the homozygous non-reference SNPs were associated with increased susceptibility (AG genotype higher frequency of susceptible than RR or GA genotypes). The homozygous non-reference increases susceptibility by 19% in the females and 12 % in the entire population.

**Table 2:** Genotype data shows an association of LRRTM4 to ascites syndrome in female REL line birds

		All				Male				Female		
Genotype	n	Sus	Res	P-val.	n	Sus	Res	*P*-val.	n	Sus	Res	*P*-val.
AG	246	45.6	54.4	0.0083	101	41.6	58.4	0.24	113	47.8	52.2	0.047
RR	426	31.0	69.0	0.13	192	27.7	72.3	0.21	157	32.7	67.3	0.83
GA	197	34.5	65.5	1.76	86	36.5	63.5	1.65	81	29.6	70.4	0.52

Data is based on SNP genotypes using a qPCR assay. Genotype is the composite of the two SNPs on chromosome 28 assayed with P1 and P2 (Table 4) (bases: 4,405,679 and 4,405,681). For each genotype the percentage of each phenotype (Sus=susceptible; Res=resistant) is presented along with the adjusted *P*-value (*P*-val.) for All samples, and each gender. Discrepancies in totals result from missing gender data for some samples. Phenotypes are Sus (susceptible) or Res (resistant)

Male broilers with homozygous non-reference genotype for intron 6 SNPs in CPQ gene have shown approximately 20% higher overall resistance [34]. To test for potential interactions between CPQ and LRRTM4, the genotype data for LRRTM4 with the genotype data for the same samples for the CPQ gene [34] were combined. The combined genotype of heterozygous for LRRTM4 and homozygous non-reference for CPQ (RRCC) genotype was significantly associated with ascites resistance in males (*P*=0.033), contrary to the LRRTM4 genotype alone. The RRCC genotype was the most abundant genotype in the samples (*n*=131). Whereas, for most combined genotypes the males were nearly 50:50, 85% of the 61 RRCC males were resistant to AS (Table 3). For CPQ genotypes alone, 72% of male homozygous non-reference males were resistant, so the combination of the two loci may increase resistance over CPQ alone. Additionally, for homozygous reference LRRTM4 (GA), the addition of a heterozygous CPQ also seems to favor resistance, but the *P*-values are not significant. In summary, the CPQ non-reference homozygous genotype confers approximately 2:1 odds on ascites resistance in males, but the addition of one non-reference allele for LRRTM4 appears to improve the odds to approximately 6:1.

**Table 3:** Combined genotype data for LRRTM4 and CPQ suggests epistatic interactions between these genes for resistance in male REL line birds

Genotype			All				Male				Female		
LRRTM4	CPQ	n	Sus	Res	P-val.	n	Sus	Res	P-val.	n	Sus	Res	P-val.
AG	TA	23	56.5	43.5	0.375	11	45.5	54.5	3.067	11	63.6	36.4	1.081
AG	YM	26	50.0	50.0	6.107	12	44.4	55.6	5.380	12	57.1	42.9	8.443
AG	CC	72	36.4	63.6	6.910	36	50.0	50.0	7.575	31	0	100.0	3.449
RR	TA	33	33.3	66.7	1.488	18	25.0	75.0	2.340	7	41.7	58.3	3.371
RR	YM	31	42.9	57.1	4.935	15	46.7	53.3	2.026	12	27.3	72.7	3.259
RR	CC	131	25.8	74.2	0.080	61	14.8	85.2	0.033	53	36.5	63.5	4.818
GA	TA	15	38.8	61.2	4.198	8	30.6	69.4	2.488	2	48.4	51.6	2.139
GA	YM	14	25.8	74.2	0.620	8	14.8	85.2	0.582	5	36.5	63.5	3.403
GA	CC	54	40.0	60.0	6.187	19	44.4	55.6	2.382	26	3.8	69.2	2.661

SNP genotype data for LRRTM4 and CPQ genes were combined and evaluated relative to distribution with respect to ascites phenotype. The CC non-reference homozygote was previously associated with resistance (35). Statistical analyses of association was based on adjusted *P*-values comparing observed counts vs predicted counts based on HWE. Genotype for LRRTM4 is as described in Table 2. Genotype for CPQ is for SNPs corresponding to bases 127,010,709 and 127,010,716 on chromosome 2 [34]

### Tissue-specific Expression Evaluation of LRRTM4

LRRTM4 gene expression for each of the homozygous genotypes was measured by reverse transcriptase qPCR (RT-qPCR) for heart, lung, liver, brain, and testis samples. TATA-binding protein (TBP) served as the reference, as TBP is recommended across multiple human tissues [39], recommended for chicken cardio-pulmonary gene expression assays [40], and we have found it to show little variation across multiple chicken tissues [41]. No expression was detected in lung, liver, brain, or testis RNA samples, but expression was observed in the heart. When comparing the level of expression of LRRTM4 between homozygous reference (ΔΔCt ± sd = 6.8 ± 0.8) and homozygous non-reference (ΔΔCt ± sd = 5.7 ± 1.0) genotypes there was no difference in expression between genotypes (*P*=0.188). The expression data indicates that LRRTM4 in broiler heart is expressed at about 0.016 (0.5^6^) the level as TBP. The RNAseq summary in NCBI for human LRRTM4 (Gene- Full Report) suggests that LRRTM4 expression is primarily restricted to the brain. Human RNAseq RPKM values for LRRTM4 are brain 5.6±1.1, heart 0.05±0.03, liver 0.033±0.012, lung 0.24±0.14, and testis 0.024±0.011. Based on the RPKM values for human TBP and LRRTM4 for these tissues, and an average ΔCt of 15.2±1.5 for TBP in the RT-qPCR analyses from REL broilers, amplification for all these tissues for LRRTM4 was expected within the 25 qPCR cycles employed. The relative expression in human heart of LRRTM4 to TBP is 0.0154 which is in agreement with the ΔΔCt values in heart for chickens. Thus, LRRTM4 expression in lung, liver, brain, and testis, is apparently much lower than the expression observed in human. Further, the two alternative alleles of LRRTM4, distinguished based on SNP differences distal to the 3’ end of the gene, do not appear to significantly differ in expression levels. Therefore, the contribution of LRRTM4 to AS more likely result from differences in the polypeptide sequences, or post-translational processes.

## Discussion

The poultry industry has fueled the affordable production of chicken by selecting for economically important traits such as growth rate, feed conversion, and meat yield. This rapid growth and muscle deposition make chicken an excellent model for human idiopathic arteriole hypertension [10]. About 3% of the fast-growing broilers develop idiopathic arteriole hypertension and exposure to stressors such as elevated dietary sodium, cold temperature [8] or high altitude [42] can increase the incidence to 20%.

Multiple investigations have estimated high heritability of AS susceptibility which led our group to pursue the genetic basis of AS [19, 20, 22, 23, 27, 35, 43, 44]. This current study, used whole-genome resequencing in the ascites research line, REL. The REL is descended from a commercial elite line in 1996. In the current study, 28 candidate regions on 13 different chromosomes were identified (Table 1). Previous GWAS studies had identified candidate regions for ascites phenotype [29–32], however, extensive genotyping and marker-assisted selection revealed minimal, or no, association with the phenotype. Therefore, these earlier candidate regions were not informative QTLs for AS (unpublished). Conversely, the first region identified from an WGR approach contained the CPQ gene on chromosome 2. Additional genotyping confirmed this region as the first verified QTL for ascites in broilers [34]. Human CPQ gene encodes a carboxypeptidase with a function in protein hydrolysis and thyroxine synthesis [45]. Dey et al. [34] showed that the non-reference homozygous genotype for CPQ has a significant association with AS resistance in REL broilers as well as some commercial lines. This is consistent with human GWAS results, where CPQ was associated with traits such as electrocardiography, hypertension, blood pressure, and heart rate (PheGenI database at NCBI).

The CPQ work led to the expansion of the WGR analyses to the rest of the assembled genome. This identified 27 additional regions for ascites phenotype (Table 1). The 28 regions include 64 genes that either reside within or are proximal to the clusters of differentially represented SNPs. The PheGenI database reveals that many of these regions have some association with hypertension, body weight, fat deposition, heart failure, or cardiac hypertrophy in humans (Fig. 2).

Further investigation of one of these regions on chromosome 22 associated with the LRRTM4 gene supported an association with AS phenotype. LRRTM4 is a member of the leucine-rich-repeat (LRR) transmembrane protein family, whose members are involved in synapse development and maintenance of the nervous system [37]. The LRRTM4 non-reference homozygote shows an association with ascites susceptibility in REL line females. Surprisingly, the combined genotypic data for CPQ and LRRTM4 revealed a male specific, epistatic interaction between the LRRTM4 heterozygote and the intron-6 non-reference homozygote of CPQ. The interaction increased the resistance in males as compared to CPQ or LRRTM4 alone. In combination, these genes can be employed in marker-assisted selection for increasing ascites resistance in the broiler industry. However, the exact mechanisms by which these genes contribute to resistance in broilers are still unknown. Studies on understanding the gene networks, downstream regulation, or interactions between the CPQ and LRRTM4 protein products in chicken are warranted.

None of the current regions reported above have appeared in any previous GWAS studies on AS [30–33]. Identification of the CPQ gene [34] and now LRRTM4 demonstrates WGR to be an effective and robust method for high-resolution mapping of QTLs for complex traits. In traditional GWAS, the panel of SNPs utilized must be pre-specified and limited in numbers. Conversely, WGR identifies the SNPs segregating in a population and can detect tens of millions of SNPs. In the REL line WGR identified more than 12 million SNPs despite the population being closed for more than 18 generations. With decreasing costs in library construction and sequencing, coupled with increasing NGS output, the WGR approach is highly cost-effective and provides far higher resolution than conventional SNP-panel-based GWAS.

## Conclusions

CPQ and LRRTM4 are gender-biased markers for ascites resistance in broilers. MAS studies are in progress to confirm the contributions of these two gene regions for ascites syndrome resistance. The additional 26 regions are available for further investigation as potential QTLs associated with ascites syndrome. The current findings may also be relevant to pursuit of the genetics underlying idiopathic pulmonary arteriole hypertension, as well as other forms of hypertension, in humans.

## Methods

### Reference genome

All genomic positions presented are relative to the May 2018 assembly of the *Gallus gallus* genome (RefSeq accession ID: GCA_000002315.5; GRCg6a).

### Bird stocks and hypobaric chamber trials

All animal procedures were approved by the University of Arkansas Institutional Animal Care and Use Committee under protocols 15039 and 15040. Birds utilized originated from a research line (REL) representing the 18^th^ generation of unselected descendants from an elite commercial broiler stock [1, 8, 35]. The REL line is maintained at the University of Arkansas Poultry Research Farm and all live animal work was performed at that facility. At hatch 600 chicks were wing-banded, and 10 μl of blood was collected via wing vein lancet puncture of chicks for DNA isolation. The birds were then challenged for six weeks using a hypobaric chamber set at 9000 ft (543 mm of Hg) above sea level [35]. Mortality data were recorded daily. All deceased birds were necropsied to determine gender and cause of death. The birds were designated ascitic/susceptible when there was excessive abdominal fluid, flaccid heart, liver lesions, and right ventricle hypertrophy (right ventricle to total ventricle; RV/TV > 0.5). At six weeks, all survivors were euthanized by cervical dislocation and necropsied for gender and ascites phenotype. Birds with normal necropsy characters were scored as resistant.

### Genomic DNA isolation and purification

Genomic DNAs were isolated using a rapid isolation protocol [46] and archived at -20 ^o^C. DNAs for next-generation sequencing were further purified by phenol-chloroform extraction, chloroform extraction, and ethanol precipitation. DNAs were quantified by Hoechst 33258 fluorescence in a GloMax (Promega Corp., Madison, WI).

### WGR of genomic DNA

Duplicate pools of equal weights of 10 DNAs from each phenotype were pooled for each gender to construct 8 total pools, which were then submitted for Next Generation Sequencing library preparation. Libraries were sequenced, 2x125 bp paired-end on an Illumina HiSeq 2500, to generate approximately 66 Gb per library. Library construction and sequencing were performed by the Research Technology Support Facility at Michigan State University (East Lansing, MI).

### Data analyses and bioinformatics

The adapter-trimmed FASTQ sequence reads were mapped onto the May 2018 chicken genome assembly (Galgal6) using SeqMan NGen (Lasergene Suite 16; DNAStar, Madison, WI). Specific settings in NGen were SNP filter high, diploid genome, whole genome pipeline, and Illumina adapter scan. All other parameters were the default settings. The pools were coded as resistant (R) or susceptible (S), male (M) or Female (F), and biological replicate pools as 1 or 2 (i.e., RM1, RM2, SM1, SM2, RF1, RF2, SF1, SF2). Separate templated alignments for each pool were used for SNP identification and tabulation in ArrayStar (Lasergene Suite). The SNP data were exported to Excel for further analyses. The difference in the averages of SNP frequencies of replicates for each phenotype and gender were calculated and exported to .seg files for viewing in Integrative Genomics Viewer 2.7.4 [36] by chromosome, aligned to the annotated genome from NCBI.

### Exonuclease assay based genotyping

Primers and probes for exonuclease genotyping (Table 4) were designed using Primer3 (http://bioinfo.ut.ee/primer3-0.4.0/primer3/), and synthesized by Integrated DNA Technologies (IDT; Coralville, IA). Conditions for qPCR exonuclease assays were optimized for each SNP locus and then used to genotype individual DNAs from additional birds phenotyped for ascites in the hypobaric chamber. DNAs used were the same as those used previously for genotyping for CPQ [34].

**Table 4:** LRRTM4 Primers and probes for genotype or expression analyses

ID	Sequence
F1	CAGCCACTGATGCAATGAGCTGTCTGAA
R1	CTCGGTASTAGCTGAAGCACGCACATC
P1	HEX-T**G**A**A**ATCTATTACCTGCATCATCTGCCT
P2	FAM-T**A**A**G**ATCTATTACCTGCATCATCTGCCT
F2	GCCCTGCACGTATACCATCT
R2	CGACTGAGTTCCAGGTTGGT

Oligonucleotide IDs designate primers as forward (F), reverse (R), or probe (P). P1 was the reference allele, with P2 as the non-reference allele. Bases in **BOLD** are the SNPs assayed

Reactions (20μl) included 1x Taq buffer (50 mM Tris-Cl pH 8.3, 1 mM MgCl_2_, 30 μg/ml BSA), 0.2 mM MgCl_2_, 0.2 mM dNTP, 0.2 μM each forward and reverse primers, 0.05 μM each probe, 2.5 units Taq polymerase, and 2 μl DNA. cycling was denaturation at 90°C 3 mins, 10 cycles of 90°C 15s, 55°C 15s, 72°C 1 min, followed by another 30 cycles of 90°C 15s, 55°C 15s, 72°C 1 min with plate read. To verify qPCR genotype calls, relevant qPCR products were purified using RapidTip (Diffinity Genomics, West Chester, Pennsylvania) and quantified by Hoechst 33258 fluorescence as above. DNAs were submitted for capillary sequencing by Eurofins MWG Operon (Louisville, KY). Sequence data (.ab1 files) were aligned to the reference Jungle Fowl sequence using SeqMan Pro software (DNASTAR) for SNP scoring.

### Gene expression analyses

Specific gene expression was assayed by RT-qPCR for heart, liver, brain, lung, and testis RNA previously extracted from broiler tissue samples [34]. Total RNA (500 ng) was added to a mastermix consisting of 1x First Strand Buffer (Promega Corp.), 0.5 μM dNTPs, 1 μM CT_23_V primer and 100 U MMLV Reverse Transcriptase (Promega Corp.). The mixtures were incubated at 50°C for 50 min and the reaction was terminated at 65°C for 5 min. The first strand cDNA was then diluted into a qPCR mixture (as described above) containing 1x EvaGreen dye (Biotium Inc., Fremont, CA). Cycling was as for exonuclease assays (above) but were followed by a high resolution melt curve from 65 to 90^o^C at 0.1^o^C steps. RT-qPCRs were run in triplicate with TATA-box binding protein (TBP) gene as the reference, and all ΔΔCt values were relative to TBP [39]. Fold change was calculated using the ΔΔCt method [47].

### Statistical methods

Genotype frequencies from exonuclease assays were separately calculated for each phenotype and gender. The expected genotype counts for each phenotype were computed from the observed genotype frequencies from the entire population. Microsoft Excel chi-square test function was performed on the count versus expected to calculate *P*-values for allele/genotype frequencies greater than 10%. *P*-values were then multiplied by the number of alleles/genotypes to generate a simple Bonferroni adjusted *P*-value. Deviation from expected was considered statistically significant where adjusted *P*-values < 0.05. Gene expression differences for alleles were based on *P*-values from Excel t-tests (one way with unequal variance).

## Data Availability

Sequence data has been deposited in NCBI under BioSample accessions SAMN07312781, SAMN07327525, SAMN07327526, SAMN07327527, SAMN07327528, SAMN07327529, SAMN07327530, and SAMN07327531.

## Abbreviations

AS: Ascites syndrome
GWAS: Genome wide association study
HWE: Hardy weinberg equilibrium
Mbp: Mega base pair
NCBI: National center for biotechnology information
PGCP: Plasma glutamate carboxy peptidase
PHS: Pulmonary hypertension syndrome
qPCR: Quantitative polymerase chain reaction
QTL: Quantitative trait locus
RT-qPCR: Real time quantitative polymerase chain reaction
RV/TV: Right ventricle to total ventricle
SNP: Single nucleotide polymorphism
WGR: Whole genome resequencing
